# Study protocol for the 10 Top Tips (10TT) Trial: Randomised controlled trial of habit-based advice for weight control in general practice

**DOI:** 10.1186/1471-2458-12-667

**Published:** 2012-08-16

**Authors:** Rebecca J Beeken, Helen Croker, Stephen Morris, Baptiste Leurent, Rumana Omar, Irwin Nazareth, Jane Wardle

**Affiliations:** 1 Department of Epidemiology & Public Health, University College London, Gower Street, London, WC1E 6BT, UK; 2 Department of Primary Care & Population Health, University College London, Gower Street, London, WC1E 6BT, UK; 3 Department of Statistical Science, University College London, Gower Street, London, WC1E 6BT, UK

**Keywords:** Obesity, Primary care, Weight control, Habits

## Abstract

**Background:**

Primary care is the first port of call for advice about weight control. There is hence a need for simple, effective interventions that can be delivered without specialist skills. We have developed such an intervention; the 10 Top Tips (10TT). This intervention was effective with respect to weight loss in a volunteer population, but has yet to be tested in primary care. The aim of this trial is therefore to test the effectiveness of the 10TT intervention in primary care, incorporating clinical outcomes and health economic analyses.

**Methods/Design:**

The trial is a two-arm, individually-randomised, controlled trial in obese (BMI ≥ 30) adults (n = 520) in primary care, comparing weight loss in patients receiving the 10TT intervention with weight loss in a control group of patients receiving usual care. The intervention is based on habit formation theory, using written materials to take people through a set of simple weight control behaviours with strategies to make them habitual; an approach that could make it more successful than others in establishing long-term behaviour change. Patients will be recruited from 14 General Practices across England. Randomisation will be through telephoning a central randomisation service using a computer-generated list of random numbers. Patients are followed up at 3, 6, 12, 18 and 24 months. The primary outcome is weight loss at 3 months, with assessment by a health professional who is blind to group allocation. Other follow-ups will be un-blinded. We will examine whether weight loss is maintained up to 24 months. We will also assess changes in the automaticity of the 10TT target behaviours and improvement in clinical markers for potential co-morbidities. Finally, we will undertake a full economic evaluation to establish cost-effectiveness in the NHS context.

**Discussion:**

If proven to be effective when delivered through primary care, 10TT could make a highly cost-effective contribution to improvements in population health.

**Trial Registration:**

ISRCTN16347068

## Background

Obesity presents an enormous public health burden [1] contributing to morbidity and mortality through Type 2 diabetes, CVD, and cancer [2], and being associated with a range of disabilities that increase use of health services [3]. It also places substantial economic costs on society [4,5]. The cross-government strategy *Healthy Weight, Healthy Lives*[6] identifies Primary Care as the ‘first port of call’ for advice about weight control, creating a need for simple, effective interventions that can be delivered by the primary care team without specialist therapeutic skills. Current non-medical treatments (e.g. cognitive behaviour therapy) require considerable expertise on the part of the practitioner and a high level of commitment from the patient; limiting their value for first-line intervention [7,8]. Maintenance of weight loss is also notoriously difficult [9].

### A novel, theory-based, self-guided, approach to weight control

Healthy ‘habits’ in the domains of diet and physical activity are the goal of most weight-loss programmes, but few if any draw explicitly on the theory of habit-formation to create healthy habits. According to psychological theory, the essential feature of habits is that they are stimulated by environmental contextual cues. Subjectively, they appear ‘automatic’, i.e. they require minimal willpower or effort [10]. Psychological research shows that repetition of a behaviour in a consistent context enables it to become automatic, and once automatic, it is more resistant to change than deliberative (intentional) behaviours [10,11]. There has been growing interest in the application of habit theory in health behaviour change, with evidence that habit strength is associated with lower dependence on deliberative/intentional processes [12-14].

In the first therapeutic application of habit theory, we developed a simple intervention promoting a set of negative energy balance behaviours. This took the form of a leaflet (called ‘*Ten Top Tips’;* 10TT) listing the target behaviours alongside advice on repetition and context-stability. Self-monitoring was recommended during the habit acquisition phase. No further clinical contact was involved. In a pilot study, outcomes and acceptability were very positive, so we carried out a small-scale, randomised controlled trial in a volunteer population [15]. The intervention produced significantly greater weight loss than the no-treatment control condition at follow up (8 weeks) using a last-observation-carried–forward (LOCF) analysis (habit group: -2.0 kg; control: -0.4 kg). Unusually for behavioural treatments, modest weight loss continued after the end of the active treatment period, reaching−3.6 kg in completers at 32 week follow-up (LOCF = −2.6 kg), with 54% (LOCF = 26%) of participants achieving the 5% weight loss associated with beneficial health effects. Weight loss appeared to be associated with increased automaticity of behaviours, supporting the idea that habit formation underpinned the effectiveness of the intervention and the maintenance of effects after the intervention period. Importantly, the intervention was rated as easy and pleasant to implement by the participants. These findings provide a strong case for testing the intervention in a full-size randomised trial in primary care and including health outcomes.

### Study Objectives

#### Primary Research Objective

The main aim of the study is to test the hypothesis that a simple weight control intervention in people with a BMI ≥ 30 based on habit-formation theory (10TT) will achieve clinically significant loss in body weight over 3 months in obese primary care patients, compared with patients placed receiving ‘usual care’.

#### Secondary Research Objectives

The main secondary research objectives are to test for differences in waist circumference, BMI and the number of people achieving a 5% reduction in weight over the trial period, and to examine whether the effects on weight loss are maintained up to 12 and 24 months. We will also explore whether the intervention leads to improvements in, and increased automaticity of, diet and physical activity behaviours at the end of the treatment period and over the 24 month follow up period. Improvements over 3 months in clinical markers for potential co-morbidities (blood pressure, total cholesterol/low-density lipoprotein (LDL) and blood glucose) will be investigated.

Exploratory analyses will look at differences between the two groups over the trial period in health-related quality of life, self-efficacy, restraint, self-regulation and social support, as well as considering whether baseline scores on these measures contribute to differences in how effective the intervention is. Potential mediators will also be explored.

Finally, at 24 months we will undertake a full economic evaluation to establish cost-efficacy in the NHS context.

## Methods/Design

**Figure 1:**
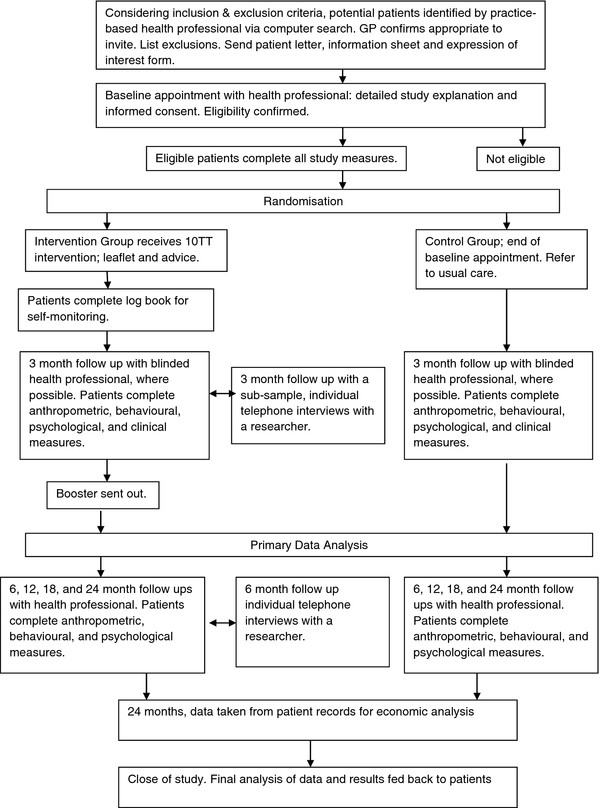
Flow chart of patient involvement in the 10TT trial.

The trial is a two-arm, individually-randomised, controlled trial in obese adults in primary care, comparing the 10TT intervention with usual care. Figure 1 illustrates the pathway through the trial.

### Recruitment

#### Participants

Obese (BMI ≥ 30) primary care patients identified from electronic General Practice records recruited from 14 General Practices.

#### Recruitment strategy/recruitment rate

Practices will be recruited through the General Practice Research Framework (GPRF) and selected to represent urban and rural areas, and to include practices serving ethnically diverse or socio-economically deprived populations. In each practice we will identify all people with recorded BMI ≥ 30 through electronic computer searches and invite them to take part. We expect BMI data to be available on 30-40% of patients, though it may have increased in recent months [16]. In practices where the number of patients with a recorded BMI ≥30 exceeds 500, a random sample of 500 patients will be selected to be invited. The GP will check the list for exclusions. Eligible patients will be sent a letter from their practice, with an enclosed information sheet, which states that the study is concerned with promoting healthy eating and activity habits to help with weight loss and that we are specifically interested in whether a leaflet describing behaviours known to be associated with weight control, along with strategies to make them habitual/automatic (‘10 Top Tips’) will lead to more weight loss compared with usual care. No further details of the intervention will be given. The letter will include an ‘expression of interest’ form on which to state whether or not they would like to take part in the study and a stamped addressed envelope. Patients may also be invited opportunistically if they have their BMI recorded after the mail-out and they are eligible. Recruitment is expected to take place over 12 months to achieve the required sample size.

#### Inclusion criteria

We will restrict the study to adults (age ≥ 18) who are able to consent for themselves, and for whom linear growth has stopped and therefore weight loss could not compromise growth. We are directing the intervention towards the obese population (BMI ≥ 30) because obesity is recognised as conferring health risk, regardless of comorbidities, and therefore the offer of treatment in the primary care context is justified.

#### Exclusion criteria

We will exclude anyone who is i) unable to provide informed consent due to mental incapacity or active psychotic illness, ii) pregnant, or iii) terminally ill. We will not have an upper age limit because we believe that interested older patients would be able to benefit.

#### Consent

Consent to enter the study will be sought from each participant after they have received an information sheet in the post beforehand and a full explanation has been given in person, with the opportunity to ask questions. The right to decline participation without giving reasons will be respected. Signed participant consent will be obtained. After the participant has entered the study their clinician remains free to give alternative treatment to that specified in the protocol at any stage if they feel it is in the participant’s best interest, but the reasons for doing so will be recorded. In these cases, participants will remain within the study for the purposes of follow-up and data analysis. All participants are free to withdraw at any time from the protocol treatment without giving reasons and without prejudicing further treatment.

### Measures

#### Demographics

Demographic data (gender, date of birth, post code, ethnicity, qualifications) will be collected at baseline.

#### Anthropometric measures

Body weight (kg) will be measured using TANITA scales supplied to practices specifically for use in this study. Patients will be asked to remove any outer garments, take off shoes and empty pockets. The usual practice equipment will be used to measure height (cm). Patients will be instructed to remove their shoes, stand with feet flat on the floor, feet together and heels against the wall, and with shoulder blades and buttocks also touching the wall, arms hanging loosely by their side, facing straight ahead. They will be instructed to breathe in deeply and stretch to their fullest height when the measurement is taken. BMI will be calculated from these measurements using the standard formula of weight (kg)/(height (m))^2^.

Waist circumference will be measured over light clothing, but patients will again be asked to remove outer layers, shoes, and tight garments. The waist is defined as the point midway between the iliac crest (top of the hip bone) and the lower rib, and measurements will be taken at the end of normal expiration.

#### Behavioural measures

Behaviour will be assessed using self-report questionnaires for diet and physical activity. Diet will be assessed using questions from the Dietary Instrument for Nutrition Education [17] on fat intake, a validated tool that has been shown to correlate with food diaries. We will also ask additional questions on fruit and vegetable intake, snacks, and type of drinks consumed (including number of units from alcohol) as these aspects of diet (along with fat intake) are specifically targeted by the intervention. We will assess physical activity using the Recent Physical Activity Questionnaire [18], a well-validated questionnaire that assesses physical activity over the past 4 weeks in 4 domains (work, travel, recreation, and domestic life). Changes in the 10TT target behaviours will be measured using 2 items that ask about the frequency of the behaviour over the past two weeks, and automaticity of the behaviours (item taken from the Self-Report Habit Index [19]).

#### Psychological measures

Brief, standardised questionnaires with good reliability and validity will be used. Health-related quality of life will be measured using the EuroQuol −5 domains (EQ-5D [20]), which asks respondents to first rate their health status on 5 domains (mobility, self-care, usual activities, pain/discomfort and anxiety/depression) and then rate their overall health state using a visual analogue scale that ranges from 0 (worst imaginable state) to 100 (best imaginable state). Self-efficacy will be assessed using the Weight Efficacy Lifestyle Questionnaire [21], a validated 20-item measure that asks participants to rate how confident they are that they can resist eating in response to five situational factors (negative emotions, availability, social pressure, physical discomfort, and positive activities). The 10-item restraint sub-scale of the Dutch Eating Behaviour Questionnaire [22] will be used to measure restrained eating. This scale has been used extensively and has strong reliability and validity. Self-regulation of eating will be assessed using a version of the Shortened Self-Regulation Questionnaire [23] adapted for the purposes of this study. The wording of the original 31 items, which assessed general self-regulation skills, was changed to apply specifically to weight regulation behaviours. For example ‘I have trouble making plans to help me reach goals’ was changed to ‘I have trouble making plans to help me reach my weight loss goals’. We also removed the ‘uncertain or unsure’ response option, so that responses are on a 4-point scale (‘strongly disagree’/‘disagree’/‘agree’/‘strongly agree’). Finally, we will assess social support for physical activity and healthy eating using brief, theory-based scales with demonstrated adequate reliability and validity [24].

#### Clinical measures

Blood pressure and blood cholesterol/LDL/glucose levels will be assessed with standard practice procedures. Blood samples are being analysed within the NHS (as opposed to by a single laboratory) so there may be some variation in assessment. Due to practical considerations we are measuring random glucose rather than fasting, which may overestimate risk in our sample.

#### Economic measures

Economic measures will include cost of the intervention, volume of resource use for primary and secondary health services (obtained from GP records) and unit costs (to be attached to the resource use data, obtained using routinely available national sources where possible and local estimates where required), and EQ-5D scores [20].

### Randomisation

The unit of randomisation is the patient. Randomisation will be done by telephoning a central randomisation service (Health Service Research Unit at Aberdeen) ensuring allocation concealment, after the participant has provided informed consent and baseline data. A computer-generated list of random permuted blocks of varying sizes will be used. Randomisation will be stratified by practice to ensure socioeconomic balance between randomised groups.

Patients randomised to the intervention group will be given the 10TT leaflet and self-monitoring log book to use in the acquisition phase, with additional brief information on the idea of forming healthy habits. Following this appointment they will be sent a booster letter with a second copy of the 10TT leaflet and can request a second log book if they would like one.

Patients randomised to the control group will be referred to the Practice’s usual care. We will ask participating Practice’s to inform us of what their usual care consists of, and this will be recorded. This will at least be a referral to a practice-based health professional not involved in the trial for a discussion on healthy eating, but in some Practices, may also be a referral to Weight Watchers.

### The intervention

10TT is a self-guided leaflet for weight management focusing on making simple diet and exercise behaviours habitual (see [15]). It is the first behaviour change intervention to be explicitly based on habit-formation theory. The component energy balance behaviours that are intended to become habitual reflected the consensus among researchers, clinicians and policy makers on healthy diet and lifestyle and were developed with input from these groups. They were also selected as practical on the basis that they were relatively common in the general population.

Table 1 outlines the tips along with their estimated calorie deficit or purpose. Seven of the 10 tips are the energy balance behaviours (intended to become habitual); three help to promote habit formation, nutrition awareness and avoidance of slips. Each ‘tip’ has a memorable name, an explanation of why it helps weight control, and suggestions on incorporating it into daily activities. Participants are also provided with a simple logbook for self-monitoring during the habit acquisition phase and a wallet sized card with guidance on food labels.

**Table 1:** **Scientific justification for the ‘10 Top Tips*’***

**Tip**	**Scientific justification**	**Estimated daily calorie deficit**
**1. Keep to your meal routine**		
Try to eat at roughly the same times each day, whether this is two or five times a day.	People who succeed at long term weight loss tend to have a regular meal rhythm (avoidance of snacking and nibbling) and show ‘flexible’ rather than ‘rigid’ control’ of eating [25]. A consistent diet regimen across the week and year also predicts subsequent long-term weight loss maintenance [26].	This tip helps encourage habit development.
**2. Go reduced fat**		
Choose reduced fat foods (e.g. dairy foods, spreads, salad dressings) where you can. Use high fat foods (e.g. butter and oils) sparingly, if at all.	There is a great deal of evidence to support the effectiveness of low-fat diets (where 30 % or less of total daily energy is from fat), which produce weight loss by decreasing calorie intake [27]. Following a low-fat diet is also associated with better weight maintenance [28].	- 200 Kcal
**3. Walk off the weight**		
Walk 10,000 steps (equivalent to 60–90 minutes moderate activity) each day. You can use a pedometer to help count the steps.	Achieving the UK government recommendation of at least 30 minutes of at least moderate intensity physical activity on 5 or more days a week would increase most people’s energy expenditure and contribute to weight management [29]. More activity (45–60 mins) may be required to prevent the transition to overweight and obesity and maximize weight loss [30]. People who have lost weight may need to do 60–90 minutes of activity a day to maintain their weight loss [29,30]. Doing 10,000 steps/day is approximately the equivalent to at least 60 minutes of walking at a brisk pace (4.5 mph) [31].	- 100 to 200 Kcal
**4. Pack a healthy snack**		
If you snack, choose a healthy option such as fresh fruit or low calorie yogurts instead of chocolate or crisps.	Readily-available snack foods and drinks are often high in energy and tend to be used to supplement rather than replace meals. Between 1993 and 1998 sales of snacks more than tripled in the UK from £173 million to £541 million [4]. Snack consumption is related to a higher daily energy intake [32].	- 150 Kcal
**5. Learn the labels**		
Be careful about food claims. Check the fat and sugar content on food labels when shopping and preparing food.	Food labels detailing the caloric and nutritional content of foods provide a basis for making healthy food choices [4]. Inadequate labeling can have a negative impact on nutrition [4]. Providing individuals with simple methods to understand labels will facilitate informed choices [33].	This tip helps people to make informed choices.
**6. Caution with your portions**		
Don’t heap food on your plate (except vegetables). Think twice before having second helpings.	Portion sizes have increased in the past 30 years [34,35]. Larger portions contain more calories and can contribute to excess energy intake and weight gain. Eating satisfying portions of low-energy-dense foods can help enhance satiety and control hunger while restricting energy intake for weight management [35].	- 100 Kcal
**7. Up on your feet**		
Break up your sitting time. Stand up for ten minutes out of every hour.	Inactive people are more likely to be obese than active people [29]. Time spent in sedentary behaviors is related to overweight and obesity, independent of physical activity level [36,37]Decreasing sedentary time and increasing light–to-moderate activity may bring substantial health benefit [29,36].	- 100 Kcal
**8. Think about your drinks**		
Choose water or sugar-free squashes. Unsweetened fruit juice contains natural sugar so limit to one glass a day (200 ml/1/3 pint). Alcohol is high in calories; limit to one unit a day for women and two for men.	Intake of sugar-sweetened soft drinks has increased over the last 30 years; up by 135 % (278 kcal) in 5 years [38]. Higher consumption of sugar-sweetened beverages is associated with greater weight gain [39]. Intake of calorific drinks may lead to excess energy intake that is not compensated for elsewhere in the daily diet [39].	- 150 Kcal
**9. Focus on your food**		
Slow down. Don’t eat on the go or while watching TV. Eat at a table if possible.	More TV viewing tends to be associated with a higher calorie intake. Internal cues regulating food intake may not be as effective while distracted by the TV [40].	This tip helps place the focus on current habits and to avoid unconscious slips in behavior.
**10. Don’t forget your 5 a day**		
Eat at least 5 portions of fruit and vegetables a day.	The UK Department of Health recommends 400 g of fruit and vegetables a day. Fruits and vegetables have high nutritional quality and low energy density. Eating the recommended amount produces health benefits including reduction in the risk of cancer and coronary heart disease [41].	This tip is important for health.
**Total Calorie Deficit**		- 800 to 900 Kcal

Practice-based health professionals (nurses or health care assistants) in each centre will attend a training session run by the charity Weight Concern and the General Practice Research Framework (GPRF) to enable them to describe the intervention to patients and carry out all necessary study procedures. Health professionals will also be provided with a study manual. Deliverers of the intervention will be instructed to spend 30 minutes taking patients through the leaflet using a flip chart and defined script contained in the manual. Manualising the treatment in such a way ensures that the delivery is standardized. Quality checks will also be carried out and on-going support will be provided to practice staff where required.

The active treatment period is 3 months, defined in terms of the appropriate duration for habit formation and the period over which participants are recommended to keep self-monitoring records (logbook) to aid habit acquisition.

### *Follow up*

#### Proposed duration of follow-up

The primary end-point will be 3 months; other time points will provide information on maintenance of health benefits following the intervention. Data will be collected at baseline, 3, 6, 12, 18 and 24 months. We propose to assess maintenance up to 24 months to provide comparability with other interventions for obesity, and to be long enough for participants to have settled into a pattern of use and for the intervention to have lost its novelty. The study will end when all patients still involved in the study have attended their 24 month follow-up.

#### Rate of loss to follow-up

In the earlier efficacy trial, the follow-up rate was 75% at two months. Primary care research participants usually have good follow-up rates although it may be lower than in the efficacy trial because of the programme being offered rather than being volunteered for. Reminders and additional attempts to contact participants may have a positive effect on retention.

#### Measurement of outcomes at follow up

Table 2 summarises the measurement of outcomes at baseline and follow-up. Weight and waist circumference will be assessed at all time points, as will questionnaire-based measures. Clinical measures will be taken only at 3 months.

**Table 2:** Summary of baseline and follow-up assessments

**Measure**		**Month of treatment**				
	**Baseline**	**3 (End of active treatment phase)**	**6**	**12**	**18**	**24**
Weight	X	X	X	X	X	X
Waist circumference	X	X	X	X	X	X
Questionnaire-based measures	X	X	X	X	X	X
Blood pressure	X	X				
Blood cholesterol/LDL/glucose levels	X	X				

#### Qualitative interviews

We will conduct semi-structured individual telephone interviews with a purposive sample from the intervention group (n ~ 20) at 3 and 6 month follow-up to explore self-reported barriers to, and facilitators of, compliance with each of the ‘tips’.

### Compliance

The intervention requires little supervision or contact with health professionals, and the instructions are simple. In the pilot study, the ease of the intervention was well received by the participants, even those with fewer years of education, who compared it favourably with other weight loss programmes. Patients randomised to the intervention will be given logbooks with a simple system of recording daily tip adherence, and will be asked to return completed books to the researchers via post. This data will provide some idea of the level of compliance and will be presented descriptively.

### Methods for protecting against sources of bias

It is not possible to blind participants, but follow-up at the end of the active treatment period (3 months) will be with a health professional blind to the intervention allocation where possible. Practical reasons (e.g. unavailability of the appropriate health professional) may prohibit blinded follow ups for all patients at 3 months. Given that the primary outcome is at 3 months, and the use of pre-dominantly objective measures, it was felt that blinding was un-necessary after the 3 month follow up. A detailed analysis plan will be developed prior to the analysis and the data-analyst will remain blind to allocation until completion of the final analyses. We will limit non-response bias through maximising follow-up using reminder phone calls prior to their follow-up appointments. Number of contact attempts and reason for withdrawing from follow-up will be recorded to facilitate statistical analyses of non-response bias. Non-attenders will also be asked to provide a self-report of their weight over the phone. Selection bias arising from recruitment of patients with recorded BMI data is largely unavoidable but comparison of sample characteristics with those of the wider (obese) population obtained from Health Survey for England data will allow us to assess the extent of this bias.

## Statistics and data analysis

### Sample size and assumptions

The exploratory trial of the intervention [15] showed a difference of weight change between baseline and 8 weeks of 1.41 kg (SD = 1.9) between the intervention and control groups. As the trial population may be more heterogeneous, and the outcome is measured 4 weeks later, we powered the study to detect a mean difference in weight change of 1.0 kg, with a standard deviation of 2.5. Based on a two sample test, 132 evaluable participants per arm are required to detect such a difference with 90% power, and a significance level of 5%. This sample size has been calculated using the statistical software STATA. However, a clustering effect may occur in the intervention arm due to different health professionals delivering the intervention in each practice. Power calculation formulae for a partially clustered design have been reported [42]. Based on an average cluster size of 13 evaluable participants (those completing 3 months follow-up), and an ICC of 0.05 [43] in the intervention arm, a total of 364 evaluable patients at 3 months (26 in each of the 14 practices) would provide 92% power to detect such a difference. This should insure the power stays above 90% even with a small imbalance in cluster size between practices [44]. Allowing for 30% attrition (26% observed in the pilot study at 8 weeks), 260 participants would need to be recruited by arm, or 520 in total.

### Data analysis

Baseline characteristics will be reported by each arm using descriptive statistics. A random effects linear regression model accounting for clustering by health professionals delivering the intervention in the intervention arm will be used to compare the weight change at 3 months from baseline between the two groups. This analysis will be adjusted for baseline weight which should make the statistical analysis more efficient. Random effects linear regression model will also be used to investigate the clustering effects due to practice.

Bias due to missing data will be investigated. If required, sensitivity of the results to missing data will be investigated using different approaches for handling missing data, including adjusting for predictors of missingness that are related to the outcome, multiple imputation and baseline observation carried forward. The possibility that data may not be missing not at random (MNAR) will also be considered and if necessary approaches that can handle MNAR data will be used as part of the sensitivity analysis [45]. Additionally, the scope of incorporating information on reasons for dropout in the analyses will also be explored.

Secondary outcomes will be compared between the intervention groups using appropriate regression models taking account of clustering. Subgroup analyses will investigate intervention effects by gender, age (in tertiles), baseline BMI (<35 vs. ≥35), ethnic origin (white vs. other) and deprivation (tertiles of postcode-based Index of Multiple Deprivation) as part of secondary analyses. Results from all secondary analyses will be considered as exploratory.

The primary aim of the trial is to evaluate the effectiveness of offering the intervention; therefore all analyses will be performed on an intention to treat basis. A detailed analysis plan will be prepared nearer the data analysis stage.

### Economic analyses

Economic analyses will conform to NICE’s preferred methodology [46]. The costs of the intervention will be calculated using a bottom-up method. Within-trial quality adjusted life years (QALYs) gained during the follow-up period will be derived from EQ-5D scores measured throughout the trial until two years. Predicted lifetime QALYs gained will be estimated using decision-analytic modelling to predict long-term quality adjusted survival and costs. Cost-effectiveness will be measured in terms of incremental cost per unit change in body weight and per QALY gained (using both within-trial and lifetime QALYs). Results will be subjected to simple and probabilistic sensitivity analysis.

### Analysis of qualitative interviews

All interviews will be audio recorded and transcribed. Thematic analysis will be used to identify the main themes that will form the basis of our results.

## Data storage and retention

Data management will be handled by PRIMENT Clinical Trials Unit with data being held according to Good Clinical Practice (GCP) requirements. As per GCP requirements, data will be held for a minimum of ten years from completion of the project.

## Ethical approval, research governance and data access

Ethical approval was obtained from the South East London Research Ethics Committee 2 via IRAS, (Ref No. 10/H0802/59, Approval granted 9th July 2010) with other participating centres providing site-specific approval as per normal IRAS procedures. NHS Research and Development (R&D) approval was obtained from all participating NHS Boards prior to the start of the trial. Further advice and support on governance and GCP issues will be provided by PRIMENT Clinical Trials Unit, who provide Standard Operating Procedures for tasks such as obtaining consent, managing and archiving data, access to trial data, training and how to handle breaches of GCP. The trial has been submitted to the International Standard Randomised Controlled Trials (ISRCT) and allocated the number ISRCTN16347068.

## Study/trial sponsorship

University College London is the sponsor of this trial.

## Discussion

The 10TT intervention is consistent with Foresight’s recommendations which include *promoting healthier food choices, building physical activity into our lives,* and *personalised advice and support*[2]*;* complementing preventive care with treatment for those who already have weight problems.

Obese patients have regular contact with primary care even before experiencing any specific obesity-related co-morbidities [47]; providing an opportunity for early intervention. However, weight management activities in primary care are limited and inconsistent [48], with some GPs reluctant to raise the subject of weight [49], and many lacking confidence in existing treatments [50]. The 10TT intervention addresses these issues, offering a simple, low-cost, treatment that can be delivered by primary care staff without special expertise.

Existing reviews identify a need for novel, practical, empirically supported trials with adequate follow-up [8,48,51,52]. Many ongoing or recently completed behavioural clinical trials registered with the metaregister of controlled trials (mRCT) focus on interventions with high levels of therapeutic contact (e.g. counselling, tailored interventions, variable content over time). These interventions show promise for use with patients who are willing to engage in a therapeutic programme but may not be as acceptable to those with more limited interest in weight control. The habit-formation approach could complement these by providing a low-contact alternative. Evidence of better weight loss maintenance for self-guided than professionally-led programmes [53] is also supportive of this approach.

The results from this study will assess the impact of the 10TT intervention on body weight over 3 months and will provide information on whether the weight loss is maintained up to 24 months. It will also increase understanding of whether the focus on habit formation leads to improvements in, and increased automaticity of, diet and physical activity behaviours, and if it is cost-effective in the NHS context. If proven to be effective when delivered through primary care, 10TT could make a highly cost-effective contribution to improvements in population health.

## Abbreviations

10TT: 10 Top Tips; EQ-5D: EuroQol −5 domains; GCP: Good clinical practice; GPRF: General Practice Research Framework; ISRCT: International Standard Randomised Controlled Trials; LDL: Low-density lipoprotein; LOCF: Last-observation-carried-forward; mRCT: metaregister of controlled trials; MNAR: Missing not at random; NHS: National Health Service; NICE: National Institute for Health and Clinical Excellence; QALYs: Quality adjusted life years; R&D: Research and Development.

## Competing interests

The authors declare that they have no competing interests.

## Authors’ contributions

JW, IN, HC and SM conceived and wrote the funding application. JW is the PI and grant holder. RJB, BL and RO have contributed to subsequent refinements of the design and analysis plan. RJB drafted the final protocol and is the trial manager. BL and RO are the trial statisticians. SM is the health economist for the trial. JW and HC provide expertise on obesity and weight management interventions. IN provides expertise on trials and primary care. All authors revised the manuscript, and read and approved the final version.

## Pre-publication history

The pre-publication history for this paper can be accessed here:

http://www.biomedcentral.com/1471-2458/12/667/prepub
